# Mechanical barriers and transforming growth factor beta inhibitor on epidural fibrosis in a rabbit laminectomy model

**DOI:** 10.1186/s13018-018-0781-6

**Published:** 2018-04-05

**Authors:** Juan N. Albiñana-Cunningham, Purificación Ripalda-Cemboráin, Tania Labiano, José I. Echeveste, Froilán Granero-Moltó, Matías Alfonso-Olmos

**Affiliations:** 1 Orthopaedic Surgery and Traumatology Department, Clínica Universidad de Navarra, 36 Pio XII Avenue, 31008 Pamplona, Spain; 2 Orthopaedic Surgery and Traumatology Department, Complejo Hospitalario de Navarra, 3 Irunlarrea Street, 31008 Pamplona, Spain; 3 Pathology Department, Complejo Hospitalario de Navarra, 3 Irunlarrea Street, 31008 Pamplona, Spain; 4 Pathology Department, Clínica Universidad de Navarra, 36 Pío XII Avenue, 31008 Pamplona, Spain; 5 Cell Therapy Area, Clínica Universidad de Navarra, 36 Pío XII Avenue, 31008 Pamplona, Spain

**Keywords:** Epidural fibrosis, Laminectomy, TGF-β, Biomaterials

## Abstract

**Background:**

TGF-β has been described as a mediator of fibrosis and scarring. Several studies achieved reduction in experimental scarring through the inhibition of TGF-β. Fibroblasts have been defined as the cell population originating fibrosis, blocking fibroblast invasion may impair epidural fibrosis appearance. For this purpose, biocompatible materials used as mechanical barriers and a TGF-β inhibitor peptide were evaluated in the reduction of epidural fibrosis.

**Methods:**

A L6 laminectomy was performed in 40 New Zealand white rabbits. Divided into four groups, each rabbit was assigned to receive either collagen sponge scaffold (CS group), gelatin-based gel (GCP group), P144® (iTGFβ group), or left untreated (control group). Four weeks after surgery, cell density, collagen content, and new bone formation of the scar area were determined by histomorphometry. Two experienced pathologists scored *dura mater* adhesion, scar density, and inflammatory infiltrate in a blinded manner.

**Results:**

In all groups, laminectomy site was filled with fibrous tissue and the *dura mater* presented adhesions. Only GCP group presented a significant reduction in collagen content and scar density.

**Conclusion:**

GCP treatment reduces epidural fibrosis although did not prevent *dura mater* adhesion completely.

**Electronic supplementary material:**

The online version of this article (10.1186/s13018-018-0781-6) contains supplementary material, which is available to authorized users.

## Background

After tissue injury, the tissue repair process may derive in the overproduction and deposition of extracellular matrix components forming a scar. When the scar is formed over the *dura mater*, it receives the name of epidural fibrosis [1]. Its presence makes reoperation much more difficult, increasing surgery time and risks of dural tears and nerve root injury [2]. This is one common problem associated with spinal surgery considering that the incidence of lumbar spine reoperation surgery ranges from 4 to 19% [3].

To prevent epidural fibrosis, interposition of a free fat graft is a common procedure in clinical practice, although it has the potential to cause nerve root or spinal cord compression [4]. In addition, different mechanical barriers and anti-inflammatory therapies have been used for the prevention of epidural fibrosis, with variable or limited clinical success [5, 6]. An ideal material to prevent epidural fibrosis should be able to minimize the risks of neurologic compression and not interfere in the healing of the surrounding tissue. It also should be biocompatible in order to minimize foreign body reaction and inflammatory response.

The transforming growth factor β (TGF-β) initiates a wide range of effects in different cells and tissues in the body [7]. Enhanced expression of TGF-β1 has been well demonstrated in scar tissue, especially in systemic sclerosis. Increased amounts of TGF-β1 are found in wounds that heal by scar formation as opposed to tissue regeneration. This has led to clinical efforts to block scar formation with antibodies and small molecules directed against TGF-β1 [8–10].

In order to evaluate the effect of TGF-β1 in epidural fibrosis, we applied an anti TGF-β1 synthetic peptide, P144® (Digna Biotech S.L., Madrid, Spain) to a rabbit spinal surgery model. P144 showed fibrosis reduction when used in animal models of liver fibrosis, bleomycin-induced skin sclerosis, and silicone periprosthetic fibrosis [11].

Adcon L® (Gliatech Inc., Cleveland, OH, USA) and DuraGen® (Integra Neurosciences, Plainsboro, NJ) have shown good results in preventing epidural fibrosis in other studies [12]. Adcon L®, an absorbable gel matrix made of gelatin and a carbohydrate polymer (GCP), functions as a barrier to fibroblast disappearing in 3 weeks [13]. DuraGen® a collagen sponge (CS), commonly used as a dural graft, allows infiltration of fibroblasts, which use the collagen matrix pores as a scaffold to lay down new collagen, disappearing in 6–8 weeks [14].

We developed a rabbit model of epidural fibrosis after laminectomy and assessed the effect of barrier materials Adcon L® and DuraGen® as well as a TGF-β1 blocking peptide P144® on epidural fibrosis appearance.

## Methods

### Animals

All procedures performed in studies involving animals were in accordance with the ethical standards of the University of Navarra and approved by the Experimental Animal Ethics Committee of the University of Navarra (CEEA 131/10).

Forty New Zealand white male rabbits (4–5 kg body weight), undergoing a L6 laminectomy, were used in this study. Four experimental groups were created:

- Control group. The laminectomy site was flushed with saline solution (*n* = 10).
- GCP group. Adcon L® was placed in the laminectomy site covering the laminectomy defect uniformly (*n* = 10).
- CS group. A Duragen® sheet was cut to fit the laminectomy defect and placed in the laminectomy site (*n* = 10).
- iTGFβ group. P144® peptide (Polypeptide Group, Strasbourg, France), derived from the sequence of the type III receptor of the human TGF-β (encompassing amino acids 730–743, SwissProt accession number Q03167), was placed in the laminectomy site covering the laminectomy defect uniformly (*n* = 10). The cytotoxicity of iTGFβ has been evaluated in previous animal studies with no evidence of cytotoxicity reported [15].

Any rabbit with complications due to the anesthesia or the laminectomy (e.g., dural tear, neural compression) were classified as non-eligible and excluded from the study.

### Surgical procedure

**Fig. 1:**
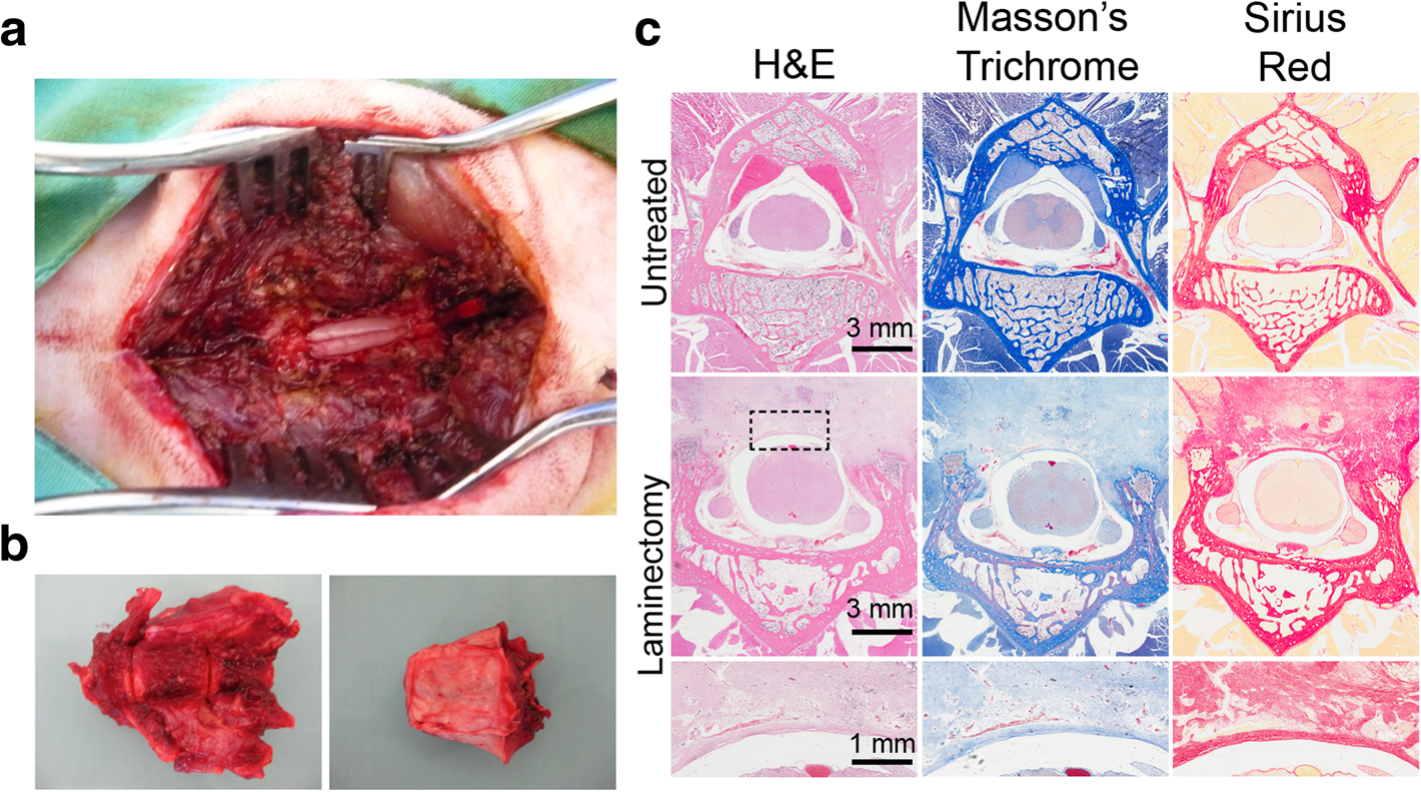
**a** Laminectomy of L6. **b** Vertebra harvesting. Left panel, ventral view; right panel, dorsal view. **c** Top panel, intact vertebrae. Middle panels, histological view of the fibrotic tissue covering the laminectomy area. Lower panel, magnification of the dural adhesion. Open box, region of the dural adhesion analyzed

Animals received a complete laminectomy of L6 up to the *ligamentum flavum* between L6 and L7 and from one pedicle to the other (Fig. 1a). All animals fasted during the 12 h previous to surgery. Animals were sedated with an intramuscular injection of medetomidine (0.15 mg/kg, Orion Pharma Espoo, Finland) and ketamine (10 mg/kg, Imalgene 1000; Merial, Lyon, France). Anesthesia was induced with an intravenous dose of propofol 2–8 mg/kg (Braun, Melsungen, Germany) and maintained with sevoflurane 1.5–3% (Abbvie, Illinois, USA) throughout the procedure. Under anesthesia, animals were placed prone on a heating pad on the operating table. The lumbosacral area was trimmed with an electric clipper and prepped with the antiseptic povidone iodine. L6 was identified by palpation and an approximated 7 cm midline incision was centered over the spinous process. The osseous plane was exposed dissecting the paraspinal musculature, the *ligamentum flavum* between L5 and L6 was reached and incised, a defect of approximately 20 mm long and 7 mm wide was then created using a 1-mm Kerrison rongeur, 45° angle, performing a complete laminectomy of L6 up to the *ligamentum flavum* between L6 and L7 and from one pedicle to the other (Fig. 1a). After treatment was applied, the fascia was closed with a 2/0 polyglactin suture (Ethicon, USA). Antibiotic was administered (penicillin/streptomycin 0.1 ml/kg/24 h) (Virbac, Esplugues de Llobregat, Spain) during 7 days. All rabbits were housed in separate cages with free access to food and water without immobilization. At 4 weeks after surgery, the rabbits were sacrificed.

All rabbits were housed in separate cages with free access to food and water without immobilization. The rabbits were sacrificed 4 weeks after surgery.

### Histology and histomorphometric analysis

For histological evaluation, the spines were harvested (Fig. 1b), fixed in 4% formalin for 1 week, and decalcified in decalcification solution (10% EDTA, 7.5% PVP, 10 mM TrisHCl pH 6.95) during 8 weeks, dehydrated in a graded ethanol, and embedded in paraffin.

Serial sections, 4 μm thick, were obtained from the midsection and from both ends of each treated level and stained with Hematoxylin and Eosin (H&E), Masson’s Trichrome, and Sirius Red dye to evaluate the scar tissue (Fig. 1c).

Digital images were acquired with a Zeiss Axiocam ICc3 camera (Plan-Neofluar objective with 0.50 NA) with an Axioimager M1 microscope (Carl Zeiss, Oberkochen, Germany).

A histomorphometric analysis was carried out quantifying cell density, new bone formation, and collagen content of the epidural scar tissue.

For cell density, the cell count was performed as cells per square millimeter using ImageJ in 15 fields per slide, 3 slides per animal. A mean number was obtained for each rabbit. Similarly, collagen content was quantified as percent of Sirius Red positive staining in the corresponding fields and mean number acquired. New bone formation area and distance covering the laminectomy expressed as area in square millimeter and distance in millimeter respectively from Masson’s Thrichrome stained sections.

Two experimented pathologists graded and scored scar density, epidural adherence, and inflammatory cell infiltrate in a blinded manner. The extension of the adhesion between *dura mater* and fibrous tissue was graded according to the classification described by He et al. [16]. Density of the adhesion was graded according to the classification used by Preul et al. [17]. The inflammatory cell infiltrate was graded with a semi quantitative scale ranging from 0 (absence of inflammatory cell infiltrate), 1 (less than 30% of the area is occupied by inflammatory cell infiltrate assessed at a × 100 magnification), 2 (moderate inflammatory cell infiltrate distributed through 30–70% of the scar tissue at a × 100 magnification), and 3 (severe inflammatory cell infiltrate distributed in over 70% of the scar tissue evident at a × 40 magnification). Each pathologist acquired three independent readings; the median of each pathologist reading was used to calculate the intra-class correlation.

### Statistical analysis

For all the statistical analysis, GraphPad Prism 5.0 software was used. The level of statistical significance was set at *p* < 0.05. Normality of continues variables were tested using Kolmogorov-Smirnov tests. Graphical data is represented as a scattered dot plot and mean value. In the text, data is shown as mean ± SD. A one-way ANOVA was used to analyze the treatment groups for differences in the mean of cell density. Dunnett’s multiple comparison test was employed to detect differences in cell density between each group with the control group. The Kruskal-Wallis test was used to analyze fibrous adherence, scar density, inflammatory infiltrate, collagen content, and new bone formation. Dunn’s multiple comparison test was used to compare the differences between each treatment group and the control group.

Intra-class agreement for all the histological scorings was analyzed using weighted Kappa coefficient.

Finally, the correlation between the % collagen and cell count was evaluated with the Spearman coefficient.

## Results

**Fig. 2:**
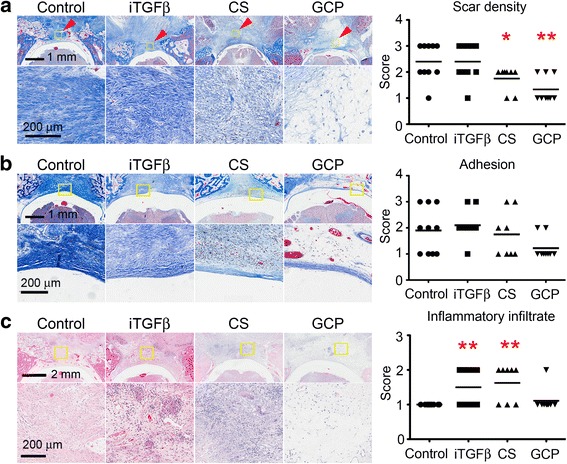
**a** Masson’s Trichrome staining evaluating scar density. Kruskal-Wallis test *p* = 0.0031; **p < 0.01. **b** Masson’s Trichrome staining evaluating *dura mater* adhesion. Kruskal-Wallis test *p* = 0.0586. **c** H&E staining evaluating inflammatory infiltrate. Kurskal-Wallis test *p* = 0.0068; *p < 0.05; **p < 0.01

To determine the effect of the different treatments in epidural fibrosis, two experimented pathologists scored scar density, *dura mater* adhesion, and inflammatory cell infiltrate. The control and iTGFβ groups presented a dense, vascularized connective tissue filling the defect resulting in a similar mean value for the scar density score (2.400 ± 0.699). In the CS group, the defect was filled with a less densely organized tissue and a significant reduction in the scar density score (1.750 ± 0.463, *p* = 0.0423). Finally, the GCP group showed the lowest scar density score, which was statistically significant when compared with the control group (1.333 ± 0.441, *p* = 0.0048) (Fig. 2a, Additional file 1: Figure S1). Intra-class agreement between observers for the scar density was 74.6% (weighted Kappa = 0.54, *p* < 0.01).

When comparing the adhesion degree between the *dura mater* and the scar tissue, we found a reduction in the score of the GCP group but without statistical significance (1.222 ± 0.441) when compared to the control group (1.900 ± 0.876, *p* = 0.0725). CS and iTGFβ groups showed no significant differences when compared with control group (1.750 ± 0.886, *p* = 0.7391 and 2.100 ± 0.567, *p* = 0.5651 respectively) (Fig. 2b, Additional file 1: Figure S1). Intra-class agreement between observers for the adhesion was calculated as 74.6% (weighted Kappa = 0.1, *p* = 0.174).

There was a significant higher score of inflammatory cell infiltrate in the iTGFβ (1.500 ± 0.527) and CS (1.625 ± 0.517) groups in comparison with the control group (1.000 ± 0.000) (*p* = 0.0119 and *p* = 0.0059 respectively). GCP group (1.111 ± 0.333) did not differ significantly from the control group (*p* = 0.1930) (Fig. 2c, Additional file 1: Figure S1). Intra-class agreement between observers for the inflammatory infiltrate was 85.7% (weighted Kappa = 0.403, *p* < 0.01).

The histomorphometric analysis showed no significant differences in the cell density count and new bone formation when comparing the treatment groups CS, iTGFβ, and GCP, with the Control group (Table 1).

**Table 1:** Histomorphometric analysis of epidural fibrosis

	Cell density (cells/area)	New bone (mm^2^)	Collagen content (%)
Control	847.6 ± 333.3	8.10 ± 5.36	61.61 ± 11.18
iTGFβ	1039.0 ± 234.0	7.53 ± 5.14	56.37 ± 10.36
CS	982.8 ± 502.4	8.15 ± 5.16	51.31 ± 13.64
GPC	486.7 ± 197.8	5.01 ± 5.14	28.55 ± 12.23^a^

Kruskal-Wallis test for collagen content *p* = 0.0002

^a^*p* < 0.0001 when compared with the control group

**Fig. 3:**
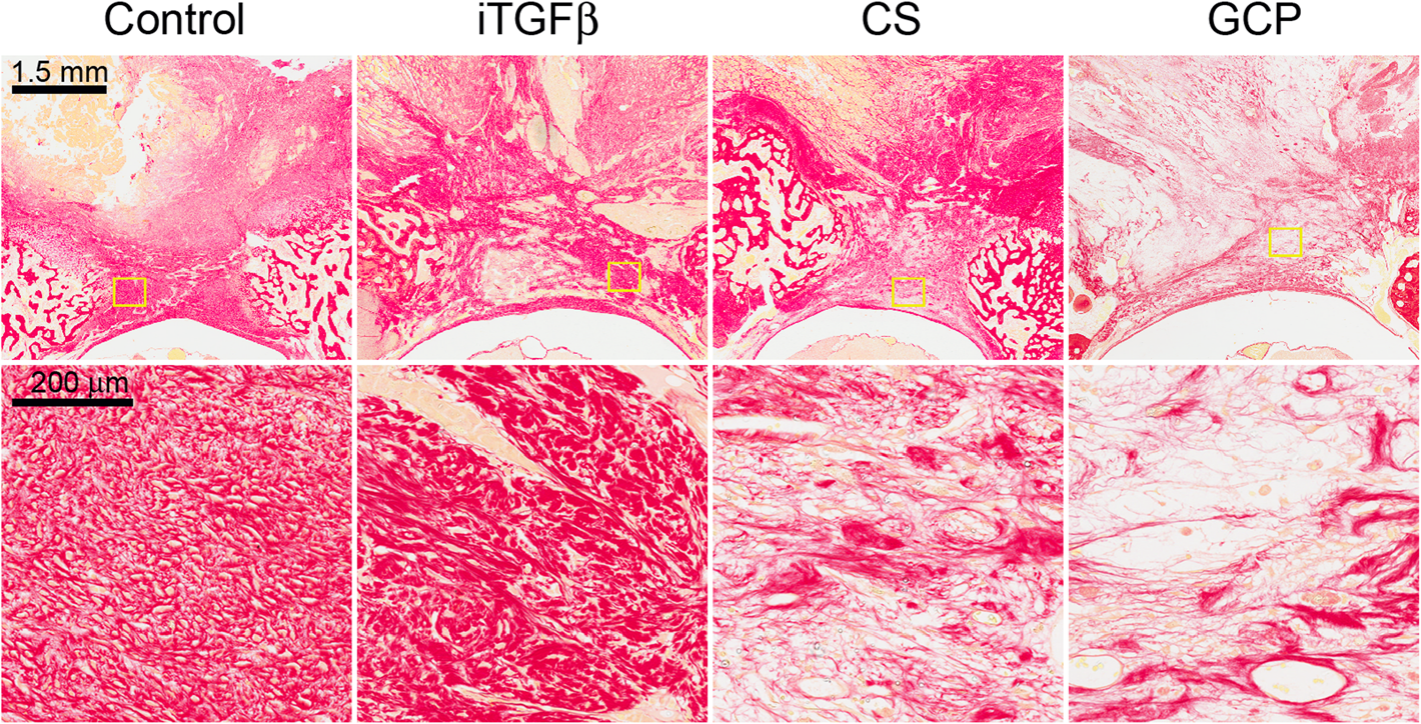
Sirius Red staining was used for histomorphometric quantification of collagen content

The collagen content was assessed by Sirius red staining. Here we did not find significant differences in the percentage of Sirius Red stained area filling the surgical site for the iTGFβ or CS treatment groups when compared to the control group (*p* = 0.6607 and *p* = 0.0545 respectively). On the other hand, a statistically significant reduction in collagen density was found in the GCP group when compared with the control group (*p* < 0.0001) (Table 1, Fig. 3).

Finally, collagen and cell content showed a significant correlation (Spearman *r* = 0.47, CI 0.17–0.70, *p* = 0.003).

## Discussion

We performed a histomorphometric study of the epidural fibrosis originated after applying different treatments in the epidural space in a rabbit laminectomy model. Histopathologic evaluation revealed that laminectomy caused significant epidural fibrosis 4 weeks after surgery. In our model, epidural fibrosis resulted in increased tissue cellularity and abundant extracellular matrix deposition. We found that collagen and cell content showed a significant correlation in contrast with the given definition of epidural fibrosis previously described as a low cellularity tissue with excessive deposition of extracellular matrix components [18].

Epidural fibrosis has been evaluated in many different animal models, being rats and rabbits the most frequently used animals in laminectomy models. Rabbits heal faster than humans, and complete closure of the laminectomy defect has been described in previous studies [3]. Using young rabbits (up to 3 kg weight), we observed complete closure of the laminectomy as soon as 4 weeks after surgery (data not shown). On the other hand, complete closure was not observed in our study with older rabbits weighing over 4 kg. New bone formation occurred at the edges of the laminectomy in all groups, and no statistical significant differences were observed.

Scoring and histomorphometric analysis indicate that GCP group presented less scar density, inflammatory infiltrate, *dura mater* adhesion, and low collagen content, suggesting that fibroblast infiltration is a key factor in the development of epidural scar. Although it has been observed that GCP scaffolds may inhibit dural healing and facilitate cerebral spinal fluid leakages from microscopic durotomies, and when mixed with autogenous bone graft could decrease bone formation, our results with GCP scaffolds are consistent with other reports on reducing peridural adhesion and lower scar density [19].

Much interest has been generated by the observation that increased amounts of TGF-β1 are found in wounds that heal by scar formation as opposed to tissue regeneration. This finding has led to clinical efforts to block scar formation with antibodies or small molecules directed against TGF-β and other pro-inflammatory mediators [20]. Ferguson et al. showed that embryonic wounds that heal without a scar have low levels of TGF-β1 and TGF-β2, low levels of platelet-derived growth factor, and high levels of TGF-β3. In addition, they experimentally mimic scar-free embryonic profile in mice, rats and pigs by neutralizing PDGF, TGF-β1, and TGF-β2 or adding exogenous TGF-β3 [20].

The treatment based in iTGFβ showed no significant differences in cell density and adhesion scores, as well as histomorphometric values to the control group. Although a higher score of inflammatory cell infiltrate was observed in comparison with the control group, we were not able to find any explanation for this finding because iTGFβ was delivered without a scaffold. This is the first study of the effect of iTGFβ on postsurgical scarring in the epidural space. All skin and muscle incisions healed within 1-week post operation, verifying that the iTGFβ gel did not cause significant adverse effects, although the safety of iTGFβ gel applications requires further investigations. The safety profile of the iTGFβ appears favorable due to its minimal local tissue response and lack of neurological deficits. Overall, our results differ to those reported in other tissues, which showed decreased scar tissue after iTGFβ treatment [11].

Collagen sponge scaffolds are commonly used as a dural substitute and its efficacy in preventing epidural fibrosis has been reported in a few studies at 8 and 20 weeks after surgery in a rabbit model [21]. In our hands, a less densely organized tissue filled the laminectomy site and a reduction in the scar density score was observed in comparison with the control group, although it did not reach statistical significance. Adhesion degree, cell density count, and collagen content showed no significant differences when compared to the control group. A significant higher score of inflammatory cell infiltrate in the CS group could be explained because the full resorption of the collagen matrix occurs 6–8 weeks after surgery; therefore, there might be inflammatory cell invasion due to a foreign body reaction.

## Conclusions

We demonstrated that peridural scarring formed in our animal model after laminectomy. GCP scaffold was able to reduce both collagenous tissue and cellularity in the epidural space after laminectomy. The other treatments (CS and iTGFβ) did not show efficacy in reducing the occurrence of epidural fibrosis or adhesion.

The data from the present study indicate that iTGFβ administrated in this manner and at this dosage is not capable of attenuating epidural fibrosis in a rabbit spinal surgery model.

## Additional file

Additional file 1: **Figure S1.** Graphical representation of the scores of the second pathologist. Scar density, Kruskal-Wallis test *p* = 0.0684. **, *p* < 0.01. *Dura mater* adhesion, Kruskal-Wallis test *p* = 0.0978. Inflammatory infiltrate, Kruskal-Wallis test *p* = 0.0058. *, *p* < 0.05. (TIFF 1203 kb)

## Abbreviations

CS: Collagen sponge
GCP: Gelatin and a carbohydrate polymer
TGF-β: Transforming growth factor beta
