# Supplementary figures and images for: Mechanical barriers and transforming growth factor beta inhibitor on epidural fibrosis in a rabbit laminectomy model
Source: J Orthop Surg Res. 2018 Apr 5;13:72. doi: 10.1186/s13018-018-0781-6 (PMC5887182; doi:10.1186/s13018-018-0781-6)

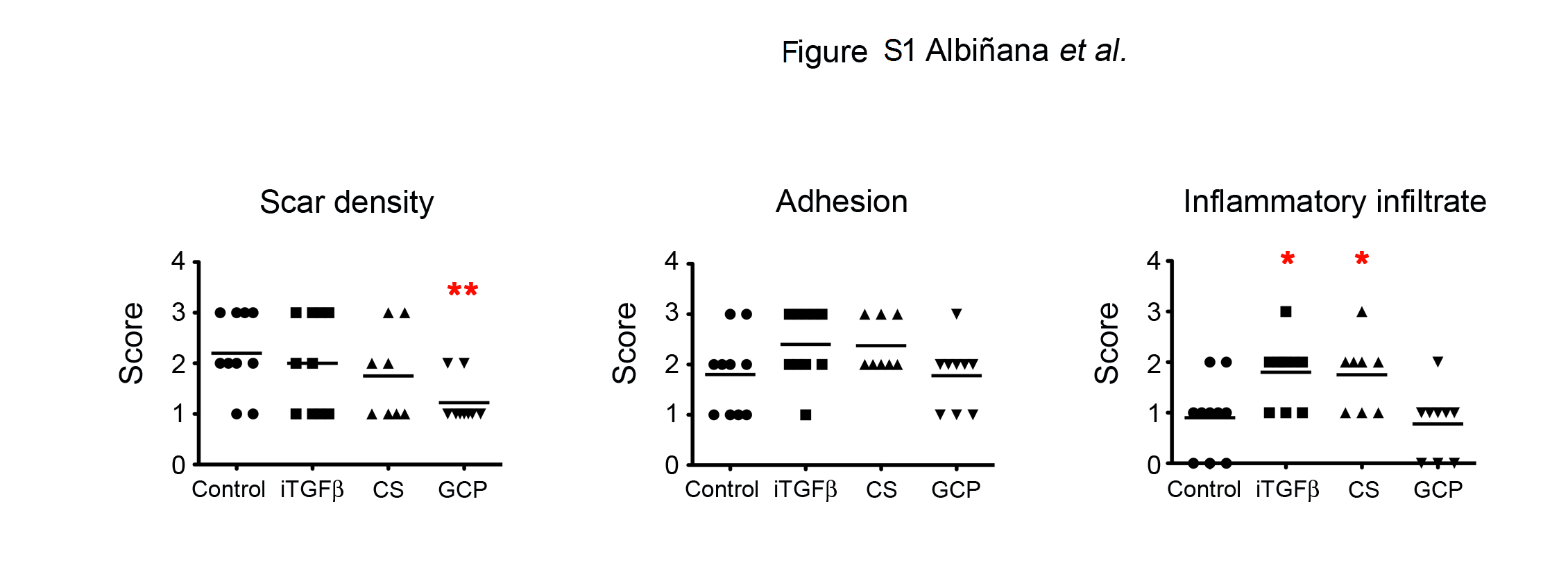

Supplement: Supplementary file 1 — Figure S1. Graphical representation of the scores of the second pathologist. Scar density, Kruskal-Wallis test p = 0.0684. **, p < 0.01. Dura mater adhesion, Kruskal-Wallis test p = 0.0978. Inflammatory infiltrate, Kruskal-Wallis test p = 0.0058. *, p < 0.05. (TIFF 1203 kb) [file 13018_2018_781_MOESM1_ESM.tif]
